# A predictive model for blastocyst formation based on morphokinetic parameters in time-lapse monitoring of embryo development

**DOI:** 10.1007/s10815-015-0440-3

**Published:** 2015-02-18

**Authors:** Robert Milewski, Paweł Kuć, Agnieszka Kuczyńska, Bożena Stankiewicz, Krzysztof Łukaszuk, Waldemar Kuczyński

**Affiliations:** 1 Department of Statistics and Medical Informatics, Medical University of Bialystok, Szpitalna 37, 15-295 Bialystok, Poland; 2 Department of Perinatology, Medical University of Bialystok, Bialystok, Poland; 3 Centre for Reproductive Medicine KRIOBANK, Bialystok, Poland; 4 Department of Reproduction and Gynecological Endocrinology, Medical University of Bialystok, Bialystok, Poland; 5 INVICTA Fertility and Reproductive Centre, Gdansk, Poland; 6 Department of Nursing, Medical University of Gdansk, Gdansk, Poland; 7 Department of Gynecology and Obstetrics, Varmia and Masuria University, Olsztyn, Poland; 8 Department of Gynecology, Medical University of Bialystok, Bialystok, Poland

**Keywords:** Infertility, IVF ET, Morphokinetic parameters, Embryoscope, Time-laps recordings

## Abstract

**Purpose:**

The aim of the study was to create a predictive model of blastocyst development based on morphokinetic parameters of time-lapse embryoscope monitoring.

**Methods:**

Time-lapse recordings of 432 embryos (obtained from 77 patients), monitored in Embryoscope, were involved in the study. Patients underwent in vitro fertilization according to standard procedure between June 2012 and April 2013. A retrospective analysis of morphokinetic features, focused on duration of time from the Intracytoplasmic Sperm Injection (ICSI) procedure to consecutive embryo division for 2, 3, 4 and 5 blastomeres, as well as time intervals between each division, was conducted. All embryos were observed for 5 days.

**Results:**

Based on the distribution of analyzed morphokinetic parameters and number of embryos developed to blastocyst, a range denoting the possibility of an embryo reaching blastocyst stage was determined. According to the obtained results, univariate and multivariate logistic regression analyses were performed. Based on the times of division for two and five blastomeres and intervals between the second and third division, a multivariate predictive model was created. The predictive equation was constructed based on the parameters of logistic regression analysis (odds ratios). Statistically significant differences (*p* < 0.001) in the size of the prediction parameter between the group of embryos developed to blastocyst (the median value: Me = 9.95, and quartiles: Q_1_ = 7.59, Q_3_ = 12.30) and embryos that did not develop to the blastocyst stage (Me = 4.66, Q_1_ = 2.33, Q_3_ = 8.19) were found. A Receiver Operating Characteristic (ROC) curve was created for the constructed predictive model. The Area Under the Curve was AUC = 0.806 with a 95 % confidence interval (0.747, 0.864). The predictive model constructed in this study has been validated using an independent data set, which indicates that the model is reliable and repeatable.

**Conclusions:**

Time-lapse imaging presents a new diagnostic tool for parametric evaluation of embryo development, from the oocyte stage, through fertilization, up to the blastocyst stage. The assessment of morphokinetic parameters can help us to provide more accurate information about the reproductive potential of embryos. It allows for early selection of embryos with high reproductive potential and shortens embryo incubation.

## Introduction

The effectiveness of Assisted Reproductive Techniques (ARTs) increases along with scientific and technological development [1]. The main goal of the In Vitro Fertilization (IVF) procedure is delivering a single healthy newborn and sending it home. In order for that process to improve, better selection as well as reduction of the number of transferred embryos is called for. Morphological assessment of embryo appearance at the proper, distinct time points during development seems to be a routine procedure in embryo selection [2, 3]. However, embryo development is a dynamic process, and some biological changes can be overlooked with standard evaluation. Moreover, good morphological features observed on the day of transfer cannot predict implantation potential [4].

Micro-cinematography offers new possibilities for noninvasive observation of early embryogenesis. The Embryoscope (Unisense Fertilitech, Aarhus, Denmark) is a combined incubator-microscope device and provides an integrated monitoring system consisting of a safe and controlled culture environment, including automated time-lapse high-resolution image acquisition [5]. The time-lapse recording intervals of a few minutes allow evaluation of embryo morphokinetic parameters such as oocyte activation, syngamy, and blastomere divisions in a continuous way. The creation of a uniform pattern of optimal time ranges for early cell divisions can allow for selection of embryos with high developmental potential [6].

Current methods of embryo evaluation need to be enhanced in order to allow for a decrease in the number of embryos transferred, without decreasing the success rate of the procedure [7]. The time-lapse technique represents a tool that provides novel information about embryo development. Some parameters observed during time-lapse recording might differ between implanting and non-implanting embryos. New markers established based on this technology could be associated with higher implantation rates. Meseguer et al. [8] conducted an analysis of retrospective data and proved that culturing embryos in the Embryoscope resulted in improved clinical pregnancy rates in an important way. The obtained benefit amounted to approximately 15 % per embryo transfer and approximately 20 % per oocyte retrieval.

Due to the possible negative impact of prolonged embryo culture on reproductive potential, early assessment of the possibility of obtaining a high quality blastocyst allows for the early selection of embryos, thereby avoiding prolonged in vitro incubation [9].

The aim of the study was to create a predictive model of blastocyst development based on morphokinetic parameters of time-lapse embryoscope monitoring.

## Materials and methods

A retrospective analysis of time-lapse recordings of 432 embryos retrieved from 77 patients (mean 5.61 embryos per patient) was performed. The study was conducted in the Centre for Reproductive Medicine Kriobank in Bialystok, Poland, between June 2012 and April 2013. All analyzed embryos were fertilized using the Intracytoplasmic Sperm Injection (ICSI) procedure.

The analysis included blastocysts in the fifth day of culture, transferred or frozen, and embryos that had not developed to the blastocyst stage. Ninety-one embryos transferred on the second or third day after fertilization were excluded from the study. Among the embryos included in the study, 156 (36.11 %) developed to the blastocyst stage, 49 (11.34 %) were transferred in fresh cycle and the remaining 107 embryos (24.77 %) were frozen. Two hundred and seventy-six embryos (63.89 %) did not reach the blastocyst stage.

The culture dish for the time-lapse process consisted of 12 cylindrical wells, each holding a culture medium droplet of 20 μl of Quinn’s Advantage Protein Plus Cleavage Medium (SAGE, USA). The droplets were covered with mineral oil (SAGE, USA) to prevent changes in medium osmolarity. Oocytes after ICSI were placed individually into wells and then slides were placed in the Embryoscope. The culture parameters were: 5.0 % CO_2_, 5.0 % O_2_ and 37.0 °C. Images of each embryo were acquired every 7 min at five different focal planes.

The absolute morphokinetic parameters (time from fertilization using ICSI to further divisions into 2,3,4 and 5 blastomeres) were presented as: t2, t3, t4 and t5, and relative morphokinetic parameters (intervals between successive divisions) as: t3-t2 (cc2) and t4-t3 (s2).

Absolute and relative morphokinetic parameters in the time-lapse monitoring of embryos developed and not developed to the blastocyst stage are presented in Table 1. Since in almost all cases (with the exception of parameter t5 in the group of embryos developed to the blastocyst stage) there was no normal distribution and in most cases a clear lack of symmetry was observed, indicators – median, quartiles, and interval (min-max) – were chosen for the description of distributions.

**Table 1:** Normality of distribution and descriptive statistics for morphokinetic parameters of embryos developed and not developed to the blastocyst stage

Parameter	Embryos developed to the blastocyst stage (h)						Embryos not developed to the blastocyst stage (h)					
	N. dist.	Min	Q_1_	Me	Q_3_	Max	N. dist.	Min	Q_1_	Me	Q_3_	Max
t2	No	19.7	24.4	26.2	28.6	40.1	No	19.8	26.7	30.1	34.5	57.4
t3	No	23.2	34.7	37.8	40.3	50.4	No	23.8	34.0	38.5	46.2	84.2
t4	No	25.8	36.0	39.2	42.3	57.6	No	26.2	36.6	42.2	50.4	84.2
t5	Yes	32.8	48.1	53.6	58.1	78.0	No	33.7	42.8	50.3	58.5	104.7
cc2	No	0.0	10.3	11.6	12.5	17.3	No	0.0	1.8	10.7	13.7	55.4
s2	No	0.00	0.00	0.67	2.00	15.9	No	0.00	0.00	0.82	8.00	40.01

Satisfactory results cannot be obtained by means of direct comparison of analyzed parameters between both groups because very high as well as very low values are not good predictors of an embryo’s ability to reach the blastocyst stage. It can be assumed that the values of the parameter most favorable to embryo development will be close to the median value in the group of embryos developed to the blastocyst stage. In order to check which parameter values correspond to the highest rates of embryo development, the values of each parameter were divided into four groups (C1-C4) relative to the first and third quartile and the median value, calculated from the entire population. This distinction provides a similar size of groups (C1-C4) designated for each of the parameters. Subsequently, for the created intervals, the percentage of embryos developed to the blastocyst stage was determined.

On the basis of the morphokinetic parameter distributions and the determined percentages of embryos developed to the blastocyst stage, an “individual score” was assigned to each C1-C4 compartment. The individual score is a parameter with a designated value of 0, 1 or 2 depending on the percentage of development in a particular compartment, and it is marked with the suffix “s_” for each morphokinetic parameter (s_t2, s_t3, etc.). Parameters of individual scores were created for subsequent development of a logistic regression model, designed to identify morphokinetic parameters affecting embryo development to the blastocyst stage.

A three-stage scale was decided upon, since evaluation using a binary scale (0–1) would not allow for a proper description of the situation where three levels (high, average and low), based on the percentage of embryos developing to the blastocyst stage, clearly appear, as in the case of division into 5 blastomeres (t5). On the other hand, a four-stage scale seemed to be too detailed because when assessed according to percentage of development, no parameters clearly showed four distinct levels. For the two compartments with the nearest percentages, the same value was assigned. However, the possibility of pairing the two middle compartments (2nd and 3rd) was excluded. The values of the individual scores assigned to each C1-C4 interval for the individual morphokinetic parameters are shown in Table 2.

**Table 2:** The individual score values assigned to quarters C1-C4 for all analyzed morphokinetic parameters

Parameter	C1		C2		C3		C4	
	% blastocysts	Individual score	% blastocysts	Individual score	% blastocysts	Individual score	% blastocysts	Individual score
t2	73.4 %	2	77.0 %	2	62.3 %	1	24.3 %	0
t3	52.9 %	0	77.6 %	2	71.4 %	1	40.9 %	0
t4	61.9 %	1	69.2 %	1	75.4 %	2	29.7 %	0
t5	46.9 %	0	63.6 %	1	79.0 %	2	62.9 %	1
cc2	39.1 %	0	75.0 %	1	85.0 %	2	43.8 %	0
s2	60.3 %*	1*	60.3 %*	1*	73.3 %	2	50.0 %	0

* parameter s2 has quarters C1 and C2 joined together

The statistical analysis of the normality distribution was verified using the Kolmogorov-Smirnov test with the Lilliefors’ amendment and the Shapiro-Wilk test. Then, univariate and multivariate logistic regression models were created. Based on the odds ratios (ORs) obtained in the multivariate logistic regression model, a prognostic parameter was built. The non-parametric U Mann–Whitney test was used to compare the values of the created parameter between two groups (according to development to the blastocyst stage). The chi-squared test of independence was conducted to compare the numbers of embryos developed to blastocyst between the studied groups. Analysis of the Receiver Operating Characteristic (ROC) with the determination of Area Under the Curve (AUC) was performed for the created predictor. Statistical significance was determined at the *p* < 0.05 level. Statistica 10.0 (StatSoft, Tulsa, OK, USA) and Stata/IC 12.1 (Stata Corp LP., College Station, TX, USA) were used in statistical analysis.

## Results

**Fig. 1:**
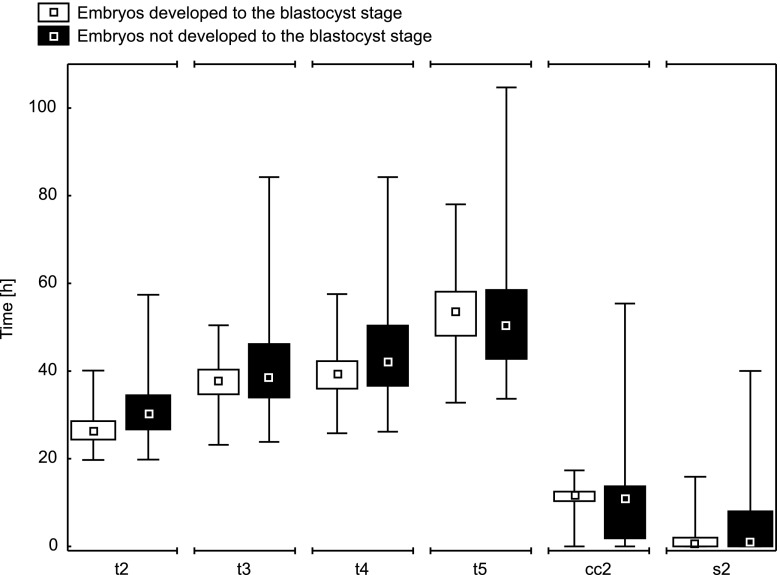
Distribution of morphokinetic parameters for embryos developed and not developed to the blastocyst stage (median, quartiles and min-max)

Assessing the distance between median values and the first and third quartiles, it can be concluded that the absolute morphokinetic parameters show greater symmetry and a smaller dispersion in the group of embryos developed to the blastocyst stage than in the remaining embryos (Fig. 1). The distributions of the analyzed time divisions in the group of embryos not developed to the blastocyst stage tend to greater dispersion, especially above the median value. The relative morphokinetic parameters (cc2 and s2) also illustrate much more dispersion in the group of embryos not developed to blastocyst.

**Fig. 2:**
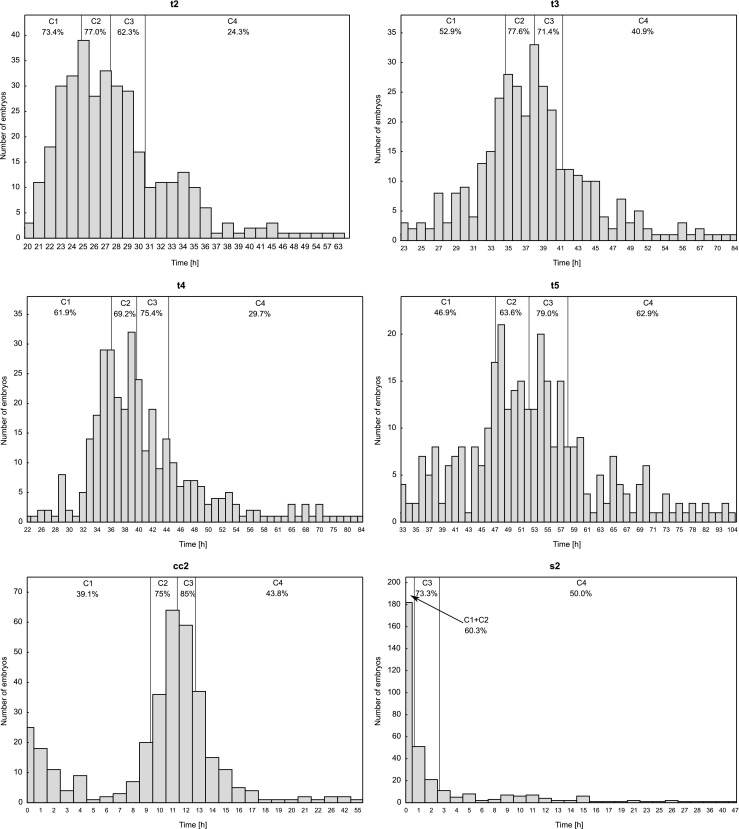
Histograms of morphokinetic parameters along with development to the blastocyst rates in groups C1-C4

Histograms for the individual parameters with selected compartments (C1-C4) and coefficients of development to the blastocyst stage are shown in Fig. 2. The s2 parameter had more than 25 % of the value equal to 0 (Min=Q_1_=0). Therefore, it was impossible to determine the C1 interval in this case because it would have a length equal to zero. Thus, for the s2 parameter in Fig. 2, the sum of the intervals C1+C2, determining the common percentage of embryos developed to blastocyst for those intervals, is presented. Embryo development was recorded every 7 min in Embryoscope. Therefore, s2 = 0 does not mean that the third and fourth blastomere divisions took place at the same time. It means that the interval between them was shorter than 7 min.

Generally, among all parameters the highest rates of development were found in compartment C3 (the average individual score was 1.67) and in compartment C2, though they were slightly lower in the latter (the C2 mean was 1.33). The lowest rates of development occurred in extreme compartments, where the average individual scores were 0.67 and 0.17 – in C1 and C4, respectively. Extreme elongation of division times (t2-t5) or the intervals between them (cc2, s2) were found to be associated with the lowest rates of development.

For the parameters of individual scores formed in this manner, univariate logistic regression analysis was performed in order to evaluate their impact on embryo development into blastocyst. Univariate logistic regression results are shown in Table 3.

**Table 3:** Univariate logistic regression analysis in relation to development to the blastocyst stage

Individual score	Odds ratio	95 % confidence interval		*p*-value
s_t2	2.947	2.115	4.105	<0.001
s_t3	2.099	1.486	2.963	<0.001
s_t4	2.783	1.871	4.142	<0.001
s_t5	2.054	1.397	3.020	<0.001
s_cc2	3.111	2.118	4.569	<0.001
s_s2	1.646	1.138	2.382	0.008

All parameters are significantly associated with the development of embryos to the blastocyst stage. t2 (OR = 2.947) presents the highest odds ratio between absolute morphokinetic parameters, and cc2 between relative morphokinetic parameters (OR = 3.111).

Taking into account the parameters whose significance was confirmed in univariate logistic regression, a multivariate logistic regression model was created (Table 4). This model takes into account the parameters s_t2, s_t5 and s_cc2.

**Table 4:** Multivariate logistic regression model in relation to development to the blastocyst stage

Individual score	Odds ratio	95 % confidence interval		*p*-value
s_t2	2.929	1.970	4.354	<0.001
s_t5	2.331	1.457	3.728	<0.001
s_cc2	2.356	1.519	3.654	<0.001

All three parameters included in the model have odds ratios higher than the value of 2.3. Taking into account the fact that the individual score is based on a three-stage scale (0–2), this means that for each of the three parameters (at constant values of the remaining components of the model) the chance of embryo development to the blastocyst stage is more than 5-fold higher in groups that have an individual score equal to two than in groups that have the individual score of zero. For the parameter s_t2 (OR = 2.929) this chance is almost nine times greater.

Based on the odds ratios determined in the multivariate logistic regression model, parameter Sc was created. It is the sum of the products of the three parameters of the model multiplied by the corresponding odds ratios. This parameter is described by the formula:

$$ \mathrm{S}\mathrm{c}=\mathrm{s}\_\mathrm{t}2*{\mathrm{OR}}_{\mathrm{s}\_\mathrm{t}2}+\mathrm{s}\_\mathrm{t}5*{\mathrm{OR}}_{\mathrm{s}\_\mathrm{t}5}+\mathrm{s}\_\mathrm{cc}2*{\mathrm{OR}}_{\mathrm{s}\_\mathrm{cc}2} $$

The higher value of the Sc parameter is determined by the higher values of individual scores taken into account in the model, while the impact of each of the individual scores on the value of Sc is proportional to the value of the odds ratio for a given parameter.

The created score seems to be a good predictor of development to the blastocyst stage. After dividing the embryos into four groups according to the median value and quartiles of the Sc parameter (C1-C4), statistically significant differences in the percentages of embryos developed to blastocyst were found between the studied groups (*p* < 0.001) (Table 5).

**Table 5:** Rates of development to the blastocyst stage between quarters of Sc parameter

Quarter (N)		C1 (55)	C2 (52)	C3 (63)	C4 (66)
Range		Sc < 5.26	5.26 ≤ Sc < 8.19	8.19 ≤ Sc < 10.54	10.54 ≤ Sc
Development to the blastocyst stage	*N*	10	35	47	59
	%	18.2 %	67.3 %	74.6 %	89.4 %

The percentage of embryos developed to the blastocyst stage increased according to Sc rise, reaching just over 18 %, in the first quarter (C1), and almost 90 % in the fourth quarter (C4).

**Fig. 3:**
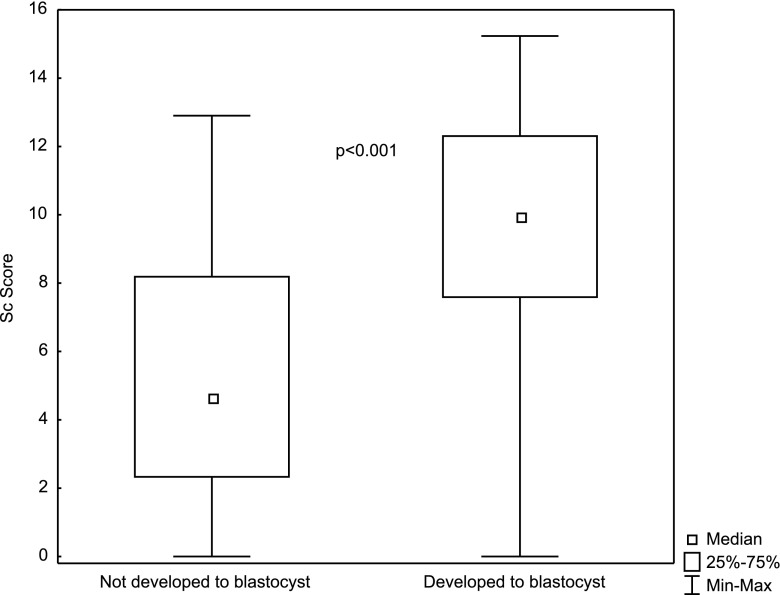
Differences in the Sc score (*p* < 0.001) between groups with and without development to the blastocyst stage (median, quartiles and min-max)

Analyzing Sc values between groups of embryos developed and not developed to the blastocyst stage also revealed statistically significant differences (*p* < 0.001). Sc values in the group of embryos developed to the blastocyst stage were significantly higher (*N* = 151; Me = 9.95; Q_1_ = 7.59; Q_3_ = 12.30) than in the group of embryos not developed to the blastocyst stage (*N* = 85; Me = 4.66; Q_1_ = 2.33; Q_3_ = 8.19). These differences are presented in Fig. 3.

**Fig. 4:**
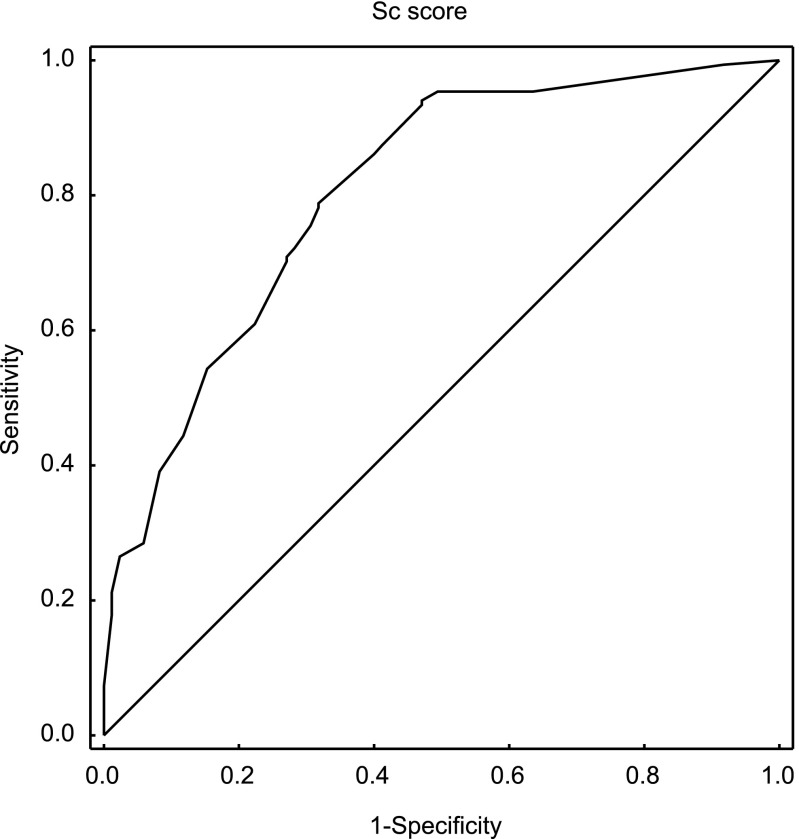
The ROC curve for prediction of development to the blastocyst stage by the Sc parameter (AUC = 0.806; 95 % CI: 0.747–0.864)

The Receiver Operating Characteristic curve created for the Sc parameter reflects the quality of this predictor as a tool for identifying the likelihood of studied embryos reaching the blastocyst stage. The ROC curve is presented in Fig. 4. The area under the ROC curve was AUC = 0.806, with a 95 % confidence interval: (0.747, 0.864). The cut-off point Sc = 7.018 was also determined using the minimal sum of squared coordinates method. Sensitivity for this cut-off point was 78.8 %, and specificity 68.2 %.

Following the conclusion of the study presented here, the created model was validated on an independent data set comprised of 271 embryos, 116 of which formed blastocysts. The results of the ROC analysis were similar: AUC = 0.813, with a 95 % confidence interval (0.746, 0.88). The cut-off point Sc = 7.59 was determined using the minimal sum of squared coordinates method. Sensitivity for this cut-off point was 75.4 %, and specificity 74.1 %. After dividing the embryos into four groups, analogously as in Table 5, statistically significant differences in the percentages of embryos developed to blastocyst were found between the studied groups (*p* < 0.001) and the obtained rates were: 26.7, 56.8, 80.0 and 90,7 % respectively. Just as presented in Fig. 3, Sc values in the group of embryos developed to the blastocyst stage were significantly higher, at the level of *p* < 0.001 (*N* = 114; Me = 9.95; Q_1_ = 7.59; Q_3_ = 12.30), than in the group of embryos not developed to the blastocyst stage (*N* = 54; Me = 5.26; Q_1_ = 2.33; Q_3_ = 7.59).

## Discussion

Proper assessment of the embryo quality at a very early phase of development is one of the most important factors affecting the success of infertility treatment using IVF methods. Montag et al. [10] claim that the scoring of early embryo development has significant limitations when based on static observations only. A time-lapse imaging system allows for revision of the scoring method, emphasizing the need for a new look at embryological factors. Lemmen et al. [11] state that the timing and coordination of events during early embryo development (from zygote to cleavage stage) are connected with embryo quality and implantation rate.

In this study, we consider only the morphokinetic parameters – absolute and relative times of division – and their impact on the ability of an embryo to achieve the blastocyst stage. This approach allows one to prove that the information contained in division time values alone allows for the creation of a helpful prediction model. Kirkegaard et al. [12] emphasize that a time-lapse monitoring system can be used to exclude embryos that would be recognized as viable using a static evaluation, but which show aberrant cleavage patterns. Therefore, further research is needed, if models based on both morphokinetic and morphological factors have a greater prediction strength than isolated morphokinetic models.

The data analyzed in the presented work has been collected on the basis of embryos cultured in the Embryoscope device. Cruz et al. [13] show that the Embryoscope system provides a proper culture environment for time-lapse imaging that does not negatively affect embryo quality or blastocyst development and viability. The main benefit of this method is the opportunity to observe developing embryos almost continuously (even at 7-min intervals).

Herrero and Meseguer [14] present some drawbacks and limitations of the time-lapse technology. For instance, the time-lapse system does not permit rotation of the embryos, making visual observation limited, in particular when blastomeres overlap or a high level of cytoplasmic fragmentation is present. In their opinion, it is doubtful whether early morphokinetic parameters (up to the five-cell stage, where embryo development is guided first of all by the maternal genome) are also representative for correct development after activation of the embryonic genome itself (from the six- to eight-cell phase and onward). Another limitation concerns constructed algorithms and working with quartiles of time ranges, which results in very exact limit points and may not be conducive to correctly predicting embryo development. In the current study, we also created a model based on quartile distribution of individual parameters, but then we created a numerical score that does not include the limitations mentioned by Herrero and Meseguer.

Meseguer et al. [6] propose using a hierarchical predictive model to identify embryos with the highest developmental potential. This model is based on morphological screening, presence or absence of exclusion criteria, timing of cell division to the five-cell phase, synchrony of divisions from the two- to four-cell phases and duration of the second cell cycle. Classification generates ten categories of embryos with increasing expected success probabilities. The hierarchical nature of this model results in the parameter considered earlier having a much greater impact on the final classification result than the parameters considered later in the hierarchy. In our model, the use of logistic regression allowed us to determine appropriate coefficients for all model parameters and to determine the final score, which had a linear rather than hierarchical form. This means that each parameter affects the final score only according to its potential in the logistic regression model, rather than depending on its position in the hierarchy.

The presented model predicts whether the embryo will develop to the blastocyst stage. Diamond et al. [15] emphasize the necessity for developing approaches that may help in the early detection of embryos capable of developing into blastocysts. This is particularly important because blastocyst transfer may not be the best solution, as it has been suggested that it brings with it an increased risk of complications in pregnancy. They propose the use of time-lapse embryo imaging for acquiring accurate data and processing it, in an attempt to obtain an algorithm that may indicate, on day 2–3, which embryos are destined for full development. Cruz et al. [16] demonstrate that kinetics of early embryo development and the potential of embryos to reach the blastocyst stage on day 5 are closely interrelated and that the use of a time-lapse monitoring system allowing for evaluation of the exact timing of early events in embryo development is a hopeful tool for the prediction of blastocyst formation and quality.

After the division of the studied data into 4 quarters according to Score parameter, the percentage of embryos reaching blastocyst stage changed from 18 % in the first quarter to almost 90 % in the fourth. Figure 3 illustrates that differences in Score distribution between embryos not developed to blastocyst and those developed to blastocyst are statistically significant; compartments 25–75 % in both groups are almost separable. The ROC analysis and the value of area under the curve AUC = 0.806 (95 % CI: 0.747–0.864) also confirmed the usefulness and predictive power of the created model.

An independent data set was also used to validate the model and the obtained results were very similar. The area under the curve was even a little bit greater (AUC = 0.813). Since validation has been achieved, this model can now be incorporated into clinical practice as a tool to support the decision-making process associated with choosing embryos to transfer on the second or third day after fertilization. There is without a doubt a need for the creation of a corresponding model to predict implantation of transferred embryos or the live birth rate of healthy children. This requires a substantial increase in the amount of collected data, because not all developing embryos, but only those selected for transfer, are taken into account in such a model.

## Conclusions

Time-lapse imaging offers completely new possibilities in the evaluation of early embryo development during the first mitotic divisions. Morphokinetic parameters allow for the creation of a model that significantly differentiates embryos that can develop to the blastocyst stage. The non-hierarchical model that we have created exhibits high predictive power and can be used in clinical practice. The estimation that an embryo has the reproductive potential to reach the blastocyst stage during the second or third day of culture allows an earlier transfer to be executed, eliminating the need for in vitro culturing with a duration of 5 to 6 days. This reduces the risk of potential complications in pregnancy.
